# Female Sex Hormones Upregulate the Replication Activity of HIV-1 Sub-Subtype A6 and CRF02_AG but Not HIV-1 Subtype B

**DOI:** 10.3390/pathogens12070880

**Published:** 2023-06-27

**Authors:** Marina Nosik, Elena Berezhnaya, Elizaveta Bystritskaya, Irina Kiseleva, Olga Lobach, Dmitry Kireev, Oxana Svitich

**Affiliations:** 1 I.I. Mechnikov Institute of Vaccines and Sera, 105064 Moscow, Russia; elvenel@gmail.com (E.B.); lisabystritskaya@gmail.com (E.B.); iakiseleva@yandex.ru (I.K.); victoriola@yandex.ru (O.L.); svitichoa@yandex.ru (O.S.); 2 Central Research Institute of Epidemiology, 111123 Moscow, Russia; dmitkireev@yandex.ru

**Keywords:** human immunodeficiency virus (HIV), β-estradiol, progesterone, toll-like receptors (TLRs), antiretroviral drugs

## Abstract

More than 50% of all people living with HIV worldwide are women. Globally, HIV/AIDS is the leading cause of death among women aged 15 to 44. The safe and effective methods of hormonal contraception are an essential component of preventive medical care in order to reduce maternal and infant mortality. However, there is limited knowledge regarding the effect of hormones on the rate of viral replication in HIV infection, especially non-B subtypes. The goal of the present work was to study in vitro how the female hormones β-estradiol and progesterone affect the replication of the HIV-1 subtypes A6, CRF02_AG, and B. The findings show that high doses of hormones enhanced the replication of HIV-1 sub-subtype A6 by an average of 1.75 times and the recombinant variant CRF02_AG by 1.4 times but did not affect the replication of HIV-1 subtype B. No difference was detected in the expression of CCR5 and CXCR4 co-receptors on the cell surface, either in the presence or absence of hormones. However, one of the reasons for the increased viral replication could be the modulated TLRs secretion, as it was found that high doses of estradiol and progesterone upregulated, to varying degrees, the expression of TLR2 and TLR9 genes in the PBMCs of female donors infected with HIV-1 sub-subtype A6.

## 1. Introduction

Worldwide, women and girls remain the most vulnerable to HIV infection. Out of 38.4 million people living with HIV, 54% (20.7 million) are women [1]. On a global scale, women and girls accounted for 49% of new HIV infections in 2021, and in sub-Saharan Africa, they accounted for about 63% [1]. According to UNAIDS, about 4900 young women aged 15–24 are infected with HIV every week [1]. At the same time, the probability of acquiring HIV infection is two times higher for the young women than for men [2]. HIV-related diseases remain the leading cause of death in women of reproductive age [1,3]. The use of modern methods of contraception helps to mitigate the risks to women’s health associated with pregnancy, especially among adolescent girls, who are still a vulnerable group, particularly in sub-Saharan Africa. In this geographical region, 1 in 10 women aged 15 to 19 years give birth, and 1 in 5 are in an official or civil marriage [4]. Methods of contraception also reduce infant mortality; when the interval between pregnancies is less than two years, the mortality rate among newborns is 45% higher compared to that of childbirth with an interval of 2–3 years and 60% higher compared to that of childbirth with an interval of 4 or more years [5]. The World Health Organization (WHO) has stated that access to qualified medical care, family planning services, and a full range of modern methods of contraception is fundamental to ensuring the rights and well-being of women and girls [6]. WHO strongly recommends the use of hormonal contraception methods for women at a high risk of acquiring HIV infection and women living with HIV, including those receiving antiretroviral therapy (ART). WHO emphasizes that the voluntary use of combined hormonal contraception by women living with HIV who wish to prevent unwanted pregnancy is an important strategy for preventing the mother-to-child transmission of HIV infection [6].

In 2019, over 436 million women worldwide used hormonal contraception methods [7]. Hormonal contraceptives include oral pills, injectable medications, implants, vaginal rings, patches, and hormone-releasing spirals [4,6,8]. The hormones used in these contraceptives include estrogen and progestins (drugs similar to the hormone progesterone), either combined or with progestin alone. The use of different types of contraception varies depending on geographical regions, but the two most common methods are oral pills and injectable progestins, especially in low- and middle-income countries [4]. Over the past 17 years, experts have been extremely concerned about a possible increase in the risk of HIV-1 infection and transmission due to the use of the injectable contraceptive medroxyprogesterone acetate (DMPA). The results of epidemiological studies on this issue were quite contradictory. Part of the large-scale long-term observations revealed that women who used DMPA and oral contraceptives had an increased risk of HIV infection compared to women who did not use hormonal contraception [9,10,11,12,13,14,15,16]. Thus, it was shown that women who use DMPA have an approximately 40% increased risk of acquiring HIV-1 infection compared to women who did not use contraception or used non-hormonal contraception [11,17,18]. At the same time, other full-scale studies have not revealed an increased risk of HIV infection associated with the use of injectable contraceptives [19,20].

In 2019, WHO revised the guidelines on the use of contraceptives by women at a high risk of HIV infection, based on epidemiological data from the large-scale randomized clinical trial (Evidence for Contraceptive Options and HIV Outcomes—ECHO) conducted among women living in four countries on the African continent [6,21]. The methods of hormonal contraception such as intramuscular DMPA injections, copper intrauterine spirals, and levonorgestrel implants were considered safe. However, despite the updated WHO guidelines, the use of DMPA remains controversial and raises questions among specialists, since extensive clinical and laboratory data indicate that this contraceptive can still increase the risk of HIV infection [22,23,24,25,26,27,28,29,30]. A number of epidemiological studies indicate that women living with HIV-1 who use hormonal contraception, compared with HIV-positive women who do not, have a significantly higher viral load, simultaneous infection with several HIV-1 genotypes, and more accelerated CD4+T cell loss, which correlates with increased mortality among this cohort [14,31,32,33,34]. Additionally, the hormone’s fluctuation during the menstrual cycle and pregnancy should be considered, which also contributes to the hormonal background. The lowest level is observed during the follicular phase of the menstrual cycle, with the highest levels of estradiol in the pre-ovulation phase and of progesterone in the luteal phase [24]. It is hypothesized that, in the luteal phase, there exists a so-called “window of vulnerability” when the risk for HIV-1 acquisition is high due to the weakening of immune responses under the influence of female hormones [35]. There are data indicating that, compared to women in the follicular phase, women in the luteal phase have higher frequencies of cervical HIV-1 target cells [15]. Along with this, there is still quite limited knowledge about the effect of female hormones on the rate of virus replication in HIV-1 infection and the overall progression of the disease. The results of a few in vitro studies on the effect of hormones on HIV-1 replication, along with clinical data, are somewhat contradictory and relate mainly to the study of HIV-1 subtype B [36,37,38]. However, globally, subtype B accounts for only 12.1% of HIV infections, while subtype A ranks second in terms of prevalence after subtype C. About 23.3% of new HIV infections in the world are caused by subtype A and its recombinant forms [39]. HIV-1 subtype A, which includes eight sub-subtypes, and its recombinant forms are widely distributed throughout Central, Western, and Southern Africa [40,41,42]. In the countries of the African continent, subtype A and its recombinant form CRF02_AG account for 22 to 53.7% of cases of HIV infection, depending on the region [39]. In the Eastern European countries, subtype A is represented by sub-subtype A6, which accounts for 52.8% of cases of HIV infection, versus 17.4% for subtype B [39]. In the former Soviet Union countries, this indicator varies from 44.6 to 94% [43,44,45,46,47]. The recombinant form CRF02_AG is one of the most common CRFs in Europe among people from countries with high endemicity as well as non-migrant populations [48,49]. Given the steadily growing migratory flows of the world population, it is likely that HIV-1 non-B subtype variants will go beyond endemic territories and become widespread in other geographical regions. Already, 20% of new HIV infections in North America and 60% in Europe are caused by non-B subtypes [50].

The main focus of the present work was to study in vitro how the female hormones β-estradiol and progesterone affected the replication of the HIV-1 subtypes A6 and CRF02_AG. In this study, we used a model of established T-cell lines and peripheral blood mononuclear cells (PBMCs) to investigate the impact of female sex hormones on the replication of the HIV-1 subtypes A6 and CRF02_AG in comparison with subtype B.

## 2. Materials and Methods

### 2.1. Cells

The cell lines Jurkat and MT-4 (from the I.I. Mechnikov Institute of Vaccines and Sera cell culture collection) were cultured in RPMI-1640 medium supplemented with 10% FBS (Sigma-Aldrich, Darmstadt, Germany, F9665), 2 mM glutamine, 100 U/mL of penicillin, and 100 U/mL of streptomycin. Human peripheral blood mononuclear cells (PBMCs) were isolated from the blood of HIV-1 seronegative female donors using Ficoll^®^-Paque Premium (Sigma-Aldrich, Darmstadt, Germany, GE17-5442-02) density-gradient centrifugation. All female donors were of reproductive age (25–35 years), and at the time of blood collection, none were taking exogenous hormones. In order to minimize the differences between donors as much as possible, blood was drawn when all women were in the ovulatory phase of the menstrual cycle (14th day).

### 2.2. Ethical Aspects

Informed consent was obtained from all donors (>18 years old) prior to blood collection in accordance with the ethical standards of the international ethical guidelines in the field of biomedical research with human participation. The study was conducted according to the guidelines of the Declaration of Helsinki and approved by the Bio-medical Ethics Committee of the I.I. Mechnikov Institute of Vaccines and Sera, Moscow, Russia (#2/03/11/20).

### 2.3. Infection with Different HIV Subtypes

PHA-stimulated PBMCs and lymphoblastoid cells were infected with HIV-1 (0.001 TCID50/cell) subtypes: A6 (GenBank: BankIt2701146 VSMO71 OQ979188), CRF02_AG (GenBank: MH062101.1), and B (GenBank: BankIt2701146 VSMO78 OQ979189) from the I.I. Mechnikov Institute of Vaccines and Sera HIV-1 isolates panel. Prior to infection, PBMCs at a concentration of 3 million/mL were aliquoted in a medium without serum for subsequent infection with each of the three HIV-1 subtypes (in triplicate). After 2 h of T-cell/PBMC + virus incubation at 37 °C in 5% CO_2_, the cells were washed twice with 1× phosphate-buffered saline (PBS). The cell pellets were resuspended in RPMI-1640 medium supplemented with 15% FBS (Sigma-Aldrich, Darmstadt, Germany, F9665). PBMCs and T-cells were cultured for 7 days in RPMI-1640 medium supplemented with 15% FBS (Sigma-Aldrich, Darmstadt, Germany, F9665), 100 U/mL of interleukin-2 (Sigma-Aldrich, Darmstadt, Germany, H7041), 2 mM glutamine, 100 U/mL of penicillin, and 100 U/mL of streptomycin. Out of the nine PBMCs isolated from female donors and infected with various subtypes of HIV-1, high viral production was achieved in PBMCs from five donors (no measurable p24 levels or low replicative activity were detected when PBMCs from the other four donors were infected with HIV-1).

### 2.4. Hormone Concentrations

Various physiological concentrations of sex steroid hormones were used in this experiment: 250 pg/mL, 5500 pg/mL of β-estradiol, (Sigma, E2257), and 89 ng/mL, 200 ng/mL of progesterone (Sigma-Aldrich, Darmstadt, Germany, P7556). Previously, we did not find a difference between the pretreatment of cells with hormones followed by infection and the simultaneous introduction of hormones with a virus (data not shown); therefore, a scheme of the simultaneous introduction of the hormones and virus was chosen. In addition, such an application scheme allowed us to study the effect of the hormones during the exponential phase of virus reproduction. Hormone concentrations were maintained throughout the entire time of the cell culture after infection.

### 2.5. Monitoring of Virus Replication

The assessment of virus replicative activity was carried out by the quantitative determination of p24 in the cell’s supernatants on the 7th day after infection using the HIV-1 p24 ANTIGEN ELISA kit (Vector-Best, Novosibirsk, Russia). p24 concentrations were determined using a standard curve obtained with the standards provided by the manufacturer. For quantification, the culture supernatants were diluted in culture media to 1:10, 1:100, and 1:1000.

### 2.6. Flow Cytometry Analysis of CCR5 and CXCR4 Co-Receptors

Flow cytometry was used to evaluate CCR5 and CXCR4 co-receptor expression levels on the surface of uninfected PBMCs and PBMCs infected with various subtypes of HIV-1 cultured in the presence and absence of hormones. The assessment of co-receptors expression in the cells was performed on Day 7 post-infection. Uninfected PBMCs and infected PBMCs with various subtypes served as controls. The following antibody panels were used: CD195 (CCR5) FITC, clone 2D7/CCR5 (D BD Biosciences, Franklin Lakes, NJ, USA) and CD184 (CXCR4) PE, clone 12G5 (D BD Biosciences, Franklin Lakes, NJ, USA ).

### 2.7. Analysis of Toll-Like Receptor (TLR) Gene Expression

Real-time reverse transcriptase-polymerase chain reaction (rRT-PCR) was per-formed to determine the expression levels of TLR2 and TLR9 genes. RNA was extracted from the PMBCs of donors using the ExtractRNA reagent, as per the manufacturer (Evrogen, Moscow, Russia). rRT-PCR was performed using the SYBR Green Syntol kit (Syntol, Moscow, Russia) and oligonucleotide primers for TLR2, TLR9, and actin genes synthesized by Syntol (Syntol, Moscow, Russia). The specific primer sets used were as follows: for TLR2, CCAGCAAATTACCTGTGTGA (forward primer) and CCCACATCATTTTCATATAC (reverse primer); for TLR9, TGGTGTTGAAGGACAGTTCTCTC (forward primer) and CACTCGGAGGTTTCCCAGC (reverse primer). The reaction was carried out under the following conditions: 1 cycle at 95 °C for 5 min, and 40 cycles at 95 °C for 15 s and 60 °C for 50 s. The assessment of TLRs expression was performed on Day 7 post-infection. Beta-actin was used as the reference gene for the analysis of TLR2 and TLR9 genes. The 2^(−ΔΔc(t)) method was used for the statistical analysis of the obtained data. For each donor, we calculated its own relative expression of the mentioned genes with the use of beta-actin expression as a reference (approved 2^(−ΔΔc(t)) method). All the values for each case were calculated against the untreated PBL of each individual. All the bars in the graphs show the one exact value for each case. Even in the case of normalization, one unified coefficient was used in the case of each donor plus each gene plus each hormone treatment parameter.

### 2.8. Statistical Analysis

Experimental replicates were averaged. Summary statistics are reported as the mean ± SD. Unpaired two-tailed Student’s *t*-test was performed to check for significant differences with group sizes of two; ANOVA test was performed for more than two groups. A *p*-value of <0.05 was considered significant. Data analysis was performed using IBM SPSS Statistics 17.0.

## 3. Results

### 3.1. Effect of β-Estradiol and Progesterone on HIV-1 Replication in T-Cell Lines and PBMCs

**Figure 1:**
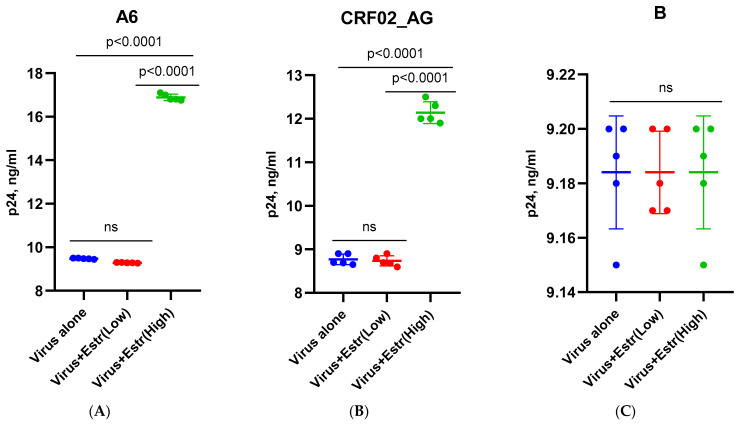

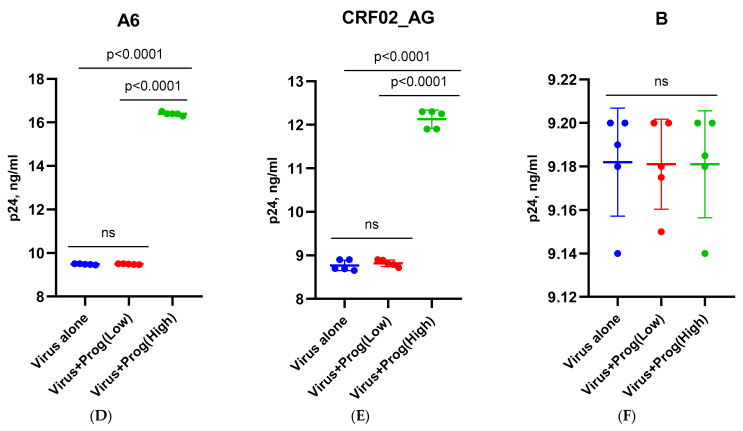
Replication of HIV-1 (sub-subtypes A6, CRF02_AG, and B) in the presence of estradiol and progesterone in MT-4 cells: low-dose β-estradiol (250 pg/mL); high-dose β-estradiol (5500 pg/mL); low-dose progesterone (89 ng/mL); high-dose progesterone (200 ng/mL). (**A**) Infection with sub-subtype A6 + β-estradiol; (**B**) infection with recombinant form CRF02_AG + β-estradiol; (**C**) infection with subtype B + β-estradiol; (**D**) infection with sub-subtype A6 + Progesterone; (**E**) infection with recombinant form CRF02_AG + Progesterone; (**F**) infection with subtype B + Progesterone. Results are representative of five independent experiments. For each experiment, all data points are the averages of three culture wells run in triplicate. For statistics, ANOVA was used.

To exclude the toxic effects of the hormones β-estradiol (at physiological concentrations of 250 and 5500 pg/mL) and progesterone (at physiological concentrations of 89 and 200 ng/mL), MT-4 and Jurkat cells and PBMCs were cultured without a virus. The studied hormone concentrations did not have a toxic effect on the cells (Supplementary Table S1). When culturing MT-4 and Jurkat cells infected with the HIV-1 subtypes A6, CRF02_AG, and B in the presence of β-estradiol at a concentration of 250 pg/mL and progesterone at a concentration of 89 ng/mL, it was found that low concentrations of hormones did not affect the reproductive activity of any HIV-1 subtype (Figure 1A–F). However, a high concentration of β-estradiol (5500 pg/mL) induced an increased replication of sub-subtype A6 and the recombinant form CRF02_AG (Figure 1A,B). In the presence of estradiol, there was an increased replication of HIV-1 sub-subtype A6 by 1.8 times (76.8%) and CRF02_AG by 1.38 times (37.9%) (*p* < 0.001 and *p* < 0.01, respectively). At the same time, the high concentration of estradiol did not affect the replication of subtype B (Figure 1C). A similar pattern of virus replication was observed in the presence of progesterone. A high concentration of progesterone (200 ng/mL) had no effect on the production of HIV-1 subtype B, whereas the production of subtypes A6 and AG increased by 1.7 times (72.6%) and 1.36 times (35.6%) (*p* < 0.001 and *p* < 0.01), respectively (Figure 1D–F). The results obtained in MT-4 and Jurkat cells were the same (Supplementary Figure S1).

**Figure 2:**
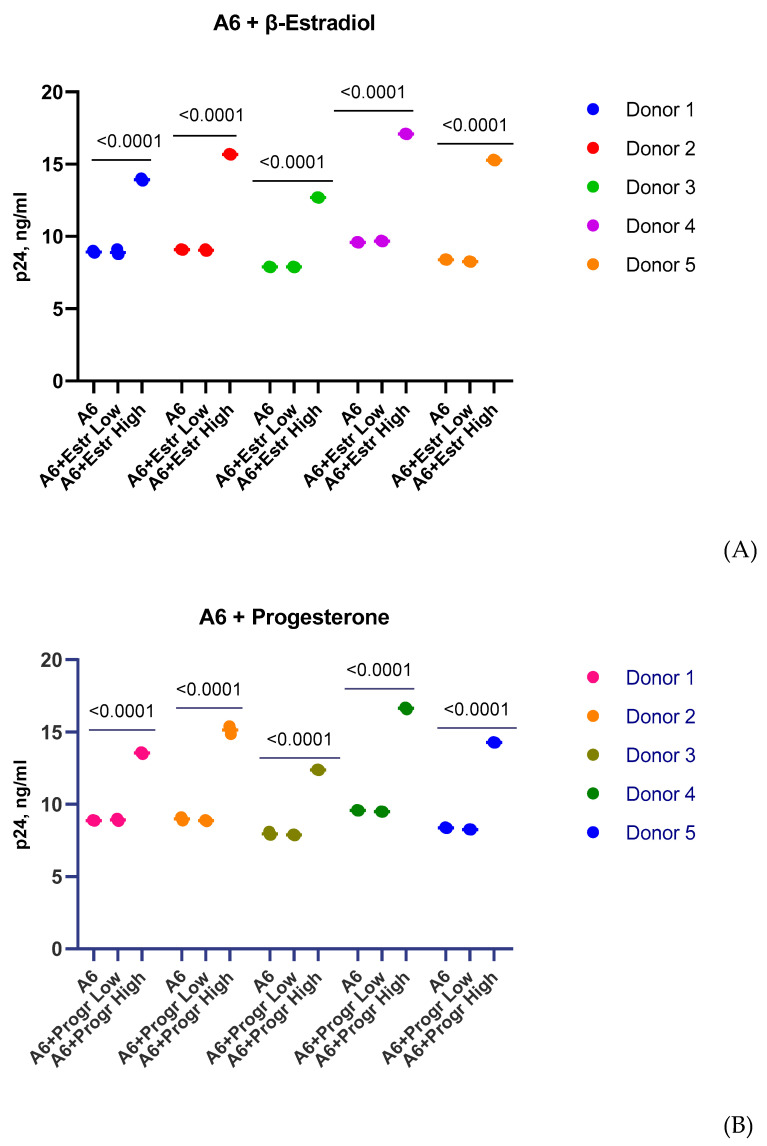

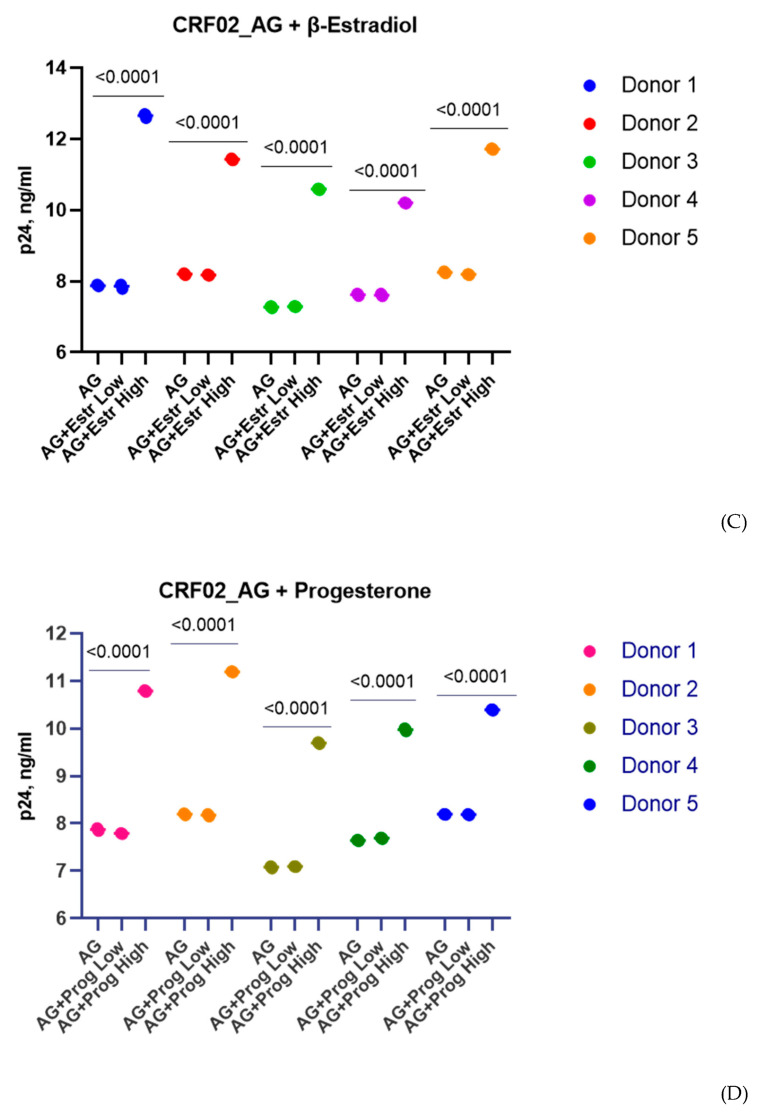

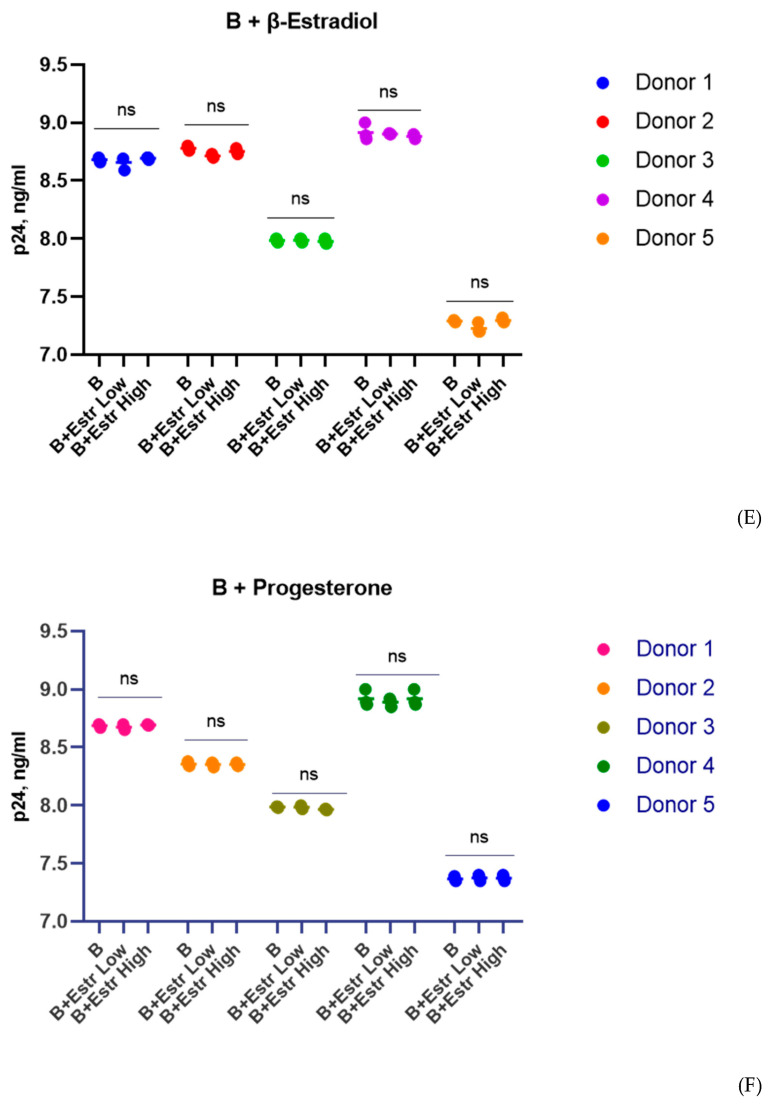
Replication of HIV-1 (sub-subtypes A6, CRF02_AG, and B) in the presence of estradiol and progesterone in the PBMCs of five female donors. Hormone concentrations: β-estradiol, 250 pg/mL (low) and 5500 pg/mL (high); progesterone, 89 ng/mL (low) and 200 ng/mL (high). (**A**) Sub-subtype A6 in the presence of estradiol. (**B**) Sub-subtype A6 in the presence of progesterone. (**C**) Recombinant form CRF02_AG in the presence of estradiol. (**D**) Recombinant form CRF02_AG in the presence of progesterone. (**E**) Subtype B in the presence of estradiol. (**F**) Subtype B in the presence of progesterone. Results are representative of three independent experiments. For each experiment, all data points are the averages of three culture wells run in triplicate; ns, no statistical difference, *p* > 0.05. For statistics, ANOVA was used.

Similar results were obtained in the donor’s PBMCs (Figure 2).

The replication activity of HIV-1 isolates in PBMCs obtained from five female donors varied slightly between donors and was comparable to the replication activity of isolates in T-cell lines (Figure 2). As with MT-4 and Jurkat cells infected with three subtypes of HIV-1 (A6, CRF02_AG, B), low doses of β-estradiol (250 pg/mL) and progesterone (89 ng/mL) had no effect on virus replication (Figure 2). In the presence of a high concentration of β-estradiol (5500 pg/mL), there was an increased production of the A6 sub-subtype by 1.6 times for Donors 1 and 3, 1.7 times for Donor 2, and 1.8 times for Donors 4 and 5 (Figure 2A). A high concentration of progesterone (200 ng/mL) enhanced virus production by 1.5 times for Donor 1, 1.6 times for Donor 3, and 1.7 times for Donors 2, 4, and 5 (Figure 2B). High concentrations of β-estradiol and progesterone also increased the replication activity of CRF02_AG: Estradiol, 1.3–1.4 times for Donors 2, 4, and 5 and 1.5–1.6 times for Donors 3 and 1; Progesterone, 1.3 times for Donors 4 and 5 and 1.4 times for Donors 1, 2, and 3 (Figure 2C,D). Thus, high concentrations of β-estradiol and progesterone upregulated the production of HIV-1 sub-subtype A6 by 3.8–3.9 lg and CRF02_AG by 3.5–3.7 lg, on average. At the same time, high doses of hormones, as well as low concentrations, did not affect the replication of HIV-1 subtype B in PBMCs (Figure 2E,F).

**Figure 3:**
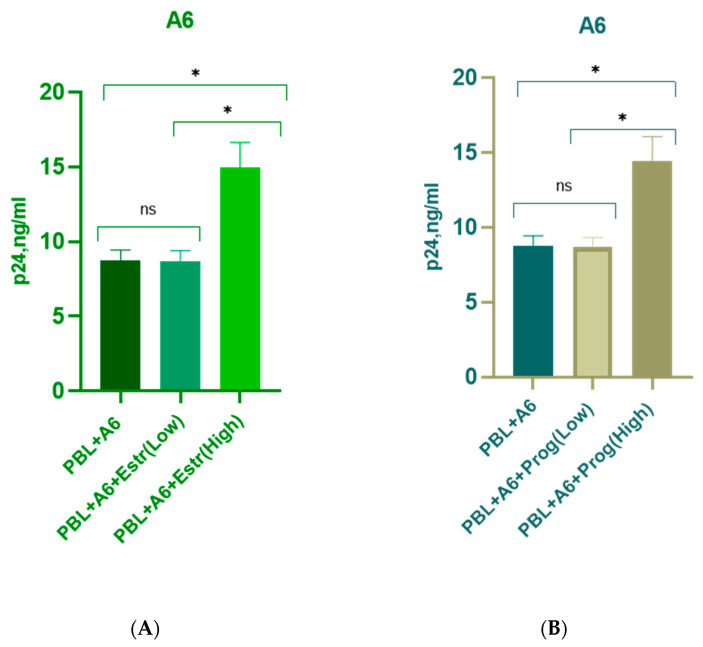

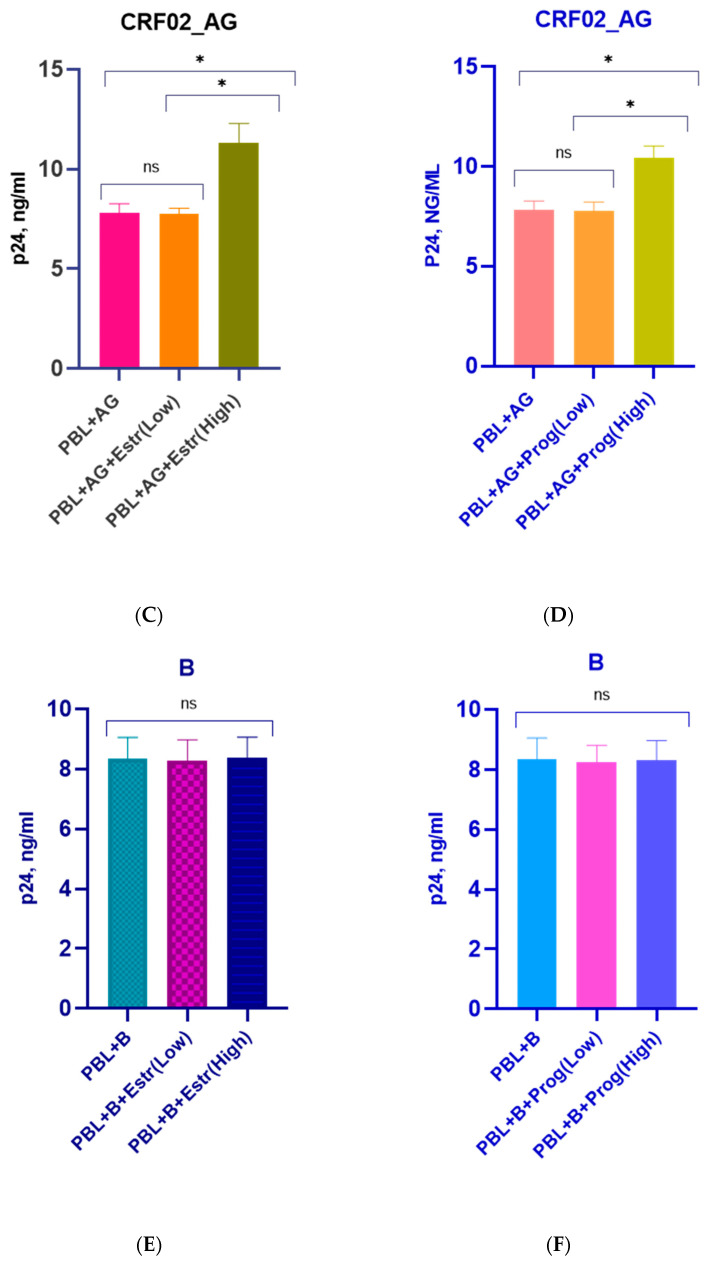
Median p24 levels in the supernatant of PBMCs (five female donors) infected with HIV-1 subtypes A6, CRF02_AG, and B and cultured in the presence of Estradiol and Progesterone. PBL, peripheral blood lymphocytes; Estr (Low)—Estradiol, 250 pg/mL; Estr (High)—Estradiol, 5500 pg/mL; Prog (low)—Progesterone, 89 ng/mL; Prog (High)—Progesterone, 200 ng/mL; (**A**) sub-subtype A6 + Estradiol; (**B**) sub-subtype A6 + Progesterone; (**C**) recombinant form CRF02_AG + Estradiol; (**D**) recombinant form CRF02_AG + Progesterone; (**E**) subtype B + Estradiol; (**F**) subtype B + Progesterone. Bars represent the mean ± SD. * *p* < 0.001; ns, no statistical difference, *p* > 0.05. For statistics, ANOVA was used.

The grouped results for all donors are presented in Figure 3.

### 3.2. Effect of β-Estradiol and Progesterone on CCR5 and CXCR4 Levels

In order to study the impact of hormones on the possible increase in the expression of CCR5 and CXCR4 co-receptors (which are known to play a key role in HIV-1 infection) on the cell surface, their expression levels were investigated in the presence/absence of steroid hormones. The assessment of co-receptors expression was performed on Day 7 post-infection. The expression levels of the co-receptors varied slightly between donors. However, there was not a significant difference in the co-receptor’s expression both in the presence of high concentrations of hormones (5500 pg/mL estradiol, 200 ng/mL progesterone) and without them. As the results were similar between PBMCs infected with HIV-1 A6 and CRF_02AG, only the data for HIV-1 A6-infected PBMCs are shown (Supplementary Figure S2).

### 3.3. Effect of β-Estradiol and Progesterone on TLR2 and TLR9 Levels

**Figure 4:**
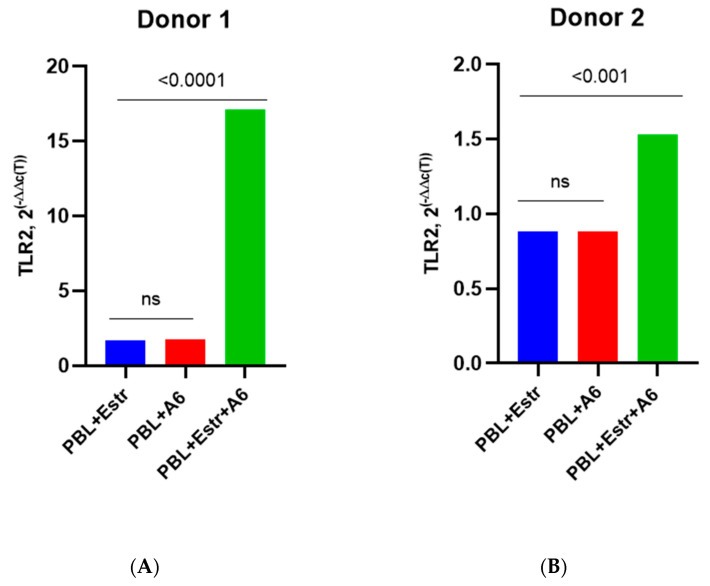

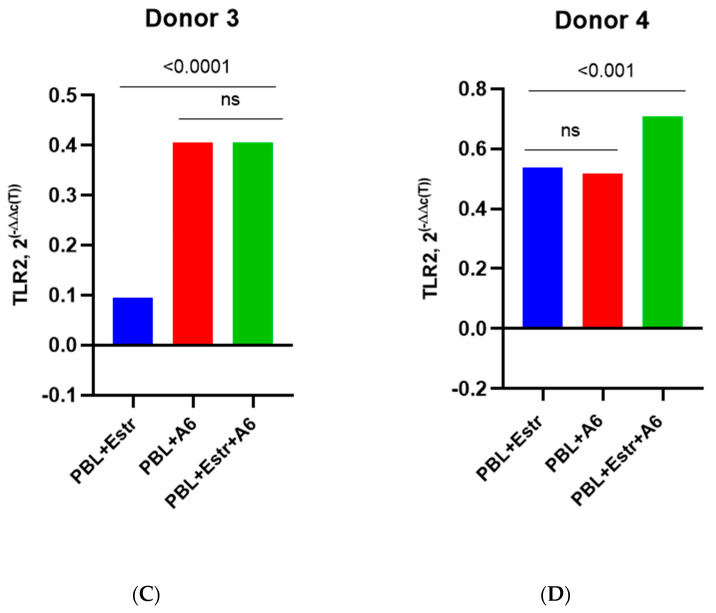
TLR2 expression in PBMCs of female donors infected by HIV-1 sub-subtype A6 in the presence of high-dose estradiol (5500 pg/mL): (**A**) Donor 1; (**B**) Donor 2; (**C**) Donor 3; (**D**) Donor 4. PBL, peripheral blood lymphocyte; Estr, estradiol; A6, HIV-1 sub-subtype A6; 2^(−∆∆C(T)), normalized expression coefficient. All data points are the averages of three culture wells run in triplicate; ns, no statistical difference, *p* > 0.05. For statistics, ANOVA was used.

Here, we present the grouped results for all four donors as well as the individual results for each donor in order to catch the similar differences. The PBMCs of four female donors infected with HIV-1 sub-subtype A6 in the presence of a high concentration of β-estradiol (5500 pg/mL) showed an increased expression of mRNA TLR2 compared to uninfected PBMCs cultured in the presence of estradiol and to HIV-1-infected PMBCs without a hormone (with the exception of Donor 3) (Figure 4). We provide data on only four donors because the samples from Donor 5 were invalid for the analysis of TLRs gene expression for technical reasons.

For Donor 1, there was an increase in mRNA TLR2 expression compared to HIV-1-infected PMBCs without a hormone by 9.8 times (*p* = 0.0001) and compared to uninfected PBMCs cultured in the presence of a hormone by 10.1 times (*p* = 0.0001) (Figure 4A). For Donors 2 and 4, there was a 1.6-fold increase in the expression of mRNA TLR2 compared to infected PMBCs without a hormone (*p* ≤ 0.0001 and *p* ≤ 0.001, respectively) (Figure 4B,D). TLR2 expression was increased compared to uninfected cells with estradiol by 1.4 times for Donor 2 and 1.3 times for Donor 4 (both *p* ≤ 0.001). The exception was Donor 3, for whom an increase in TLR2 expression in infected cells under the influence of estradiol compared with infected PBMCs without the hormone was not shown (Figure 4C). However, for Donor 3, there was enhanced expression of TLR2 in infected cells in the presence of estradiol compared to uninfected cells with estradiol, by 4.3 times (*p* < 0.0001).

**Figure 5:**
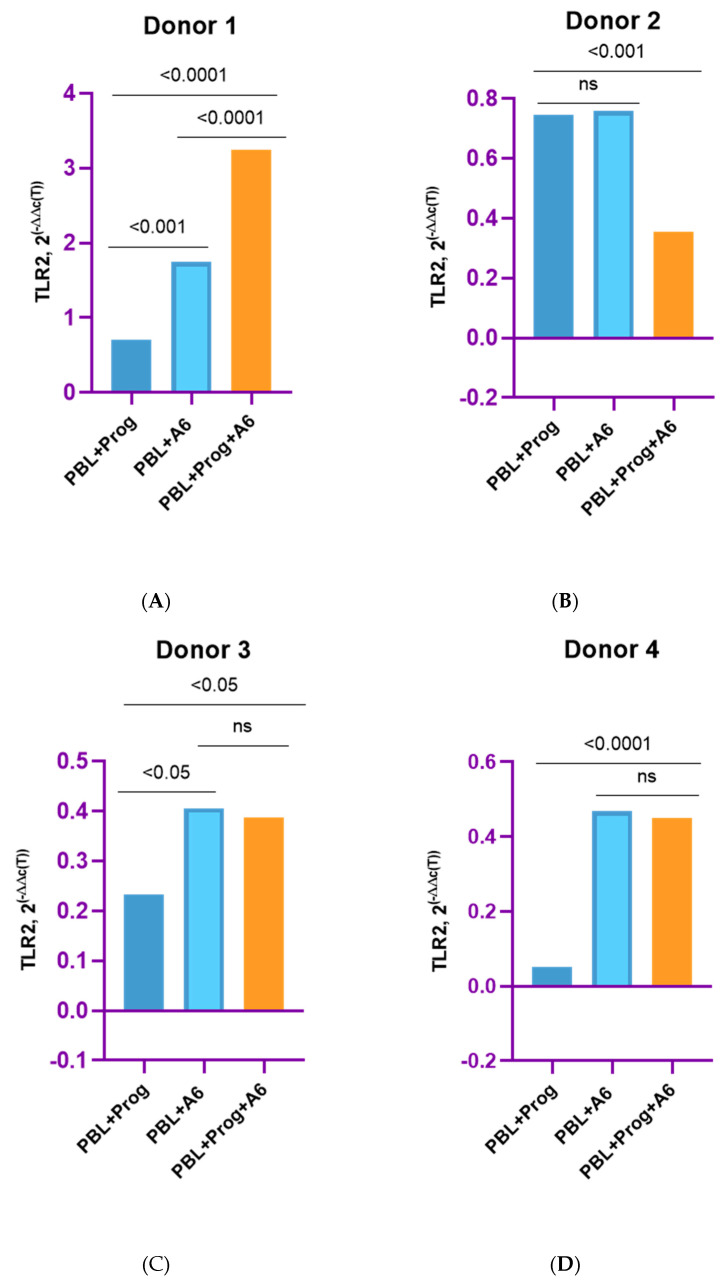
TLR2 expression in PBMCs of female donors infected with HIV-1 sub-subtype A6 in the presence of high-dose progesterone (200 ng/mL): (**A**) Donor 1; (**B**) Donor 2; (**C**) Donor 3; (**D**) Donor 4. PBL, peripheral blood lymphocyte; Prog, progesterone; A6, HIV-1 sub-subtype A6; 2^(−∆∆C(T)), normalized expression coefficient. All data points are the average of three culture wells run in triplicate; ns, no statistical difference, *p* > 0.05. For statistics, ANOVA was used.

A slightly different picture was observed when studying TLR2 expression in the presence of a high dose of progesterone (200 ng/mL) (Figure 5).

A statistically significant increase in the expression of mRNA TLR2 in infected PMBCs in the presence of progesterone was observed for Donor 1, by 1.9 times (*p* < 0.0001) compared with infected cells without the hormone and by 4.6 times (*p* < 0.001) compared with uninfected progesterone-treated lymphocytes (Figure 5A). For Donor 2, there was a twofold decrease in TLR2 expression in infected cells with progesterone vs. infected cells without a hormone (Figure 5B). For Donors 3 and 4, a slight decrease in the expression of mRNA TLR2 was shown in the presence of hormones compared to infected cells without the hormone, but these differences were not statistically significant (Figure 5C,D). However, for Donor 3, there was 1.7-fold enhanced TLR2 expression in infected PBMCs with the hormone compared to uninfected PBMCs treated with the hormone (Figure 5C). Equally, for Donor 4, a 7.6-fold increase (*p* < 0.0001) in TLR2 expression in infected cells in the presence of progesterone compared to uninfected cells treated with the hormone was shown (Figure 4D). For Donor 2, there was not a statistically significant difference in TLR2 expression between uninfected PBMCs treated with the hormone and infected PBMCs in the absence of the hormone (Figure 4B).

**Figure 6:**
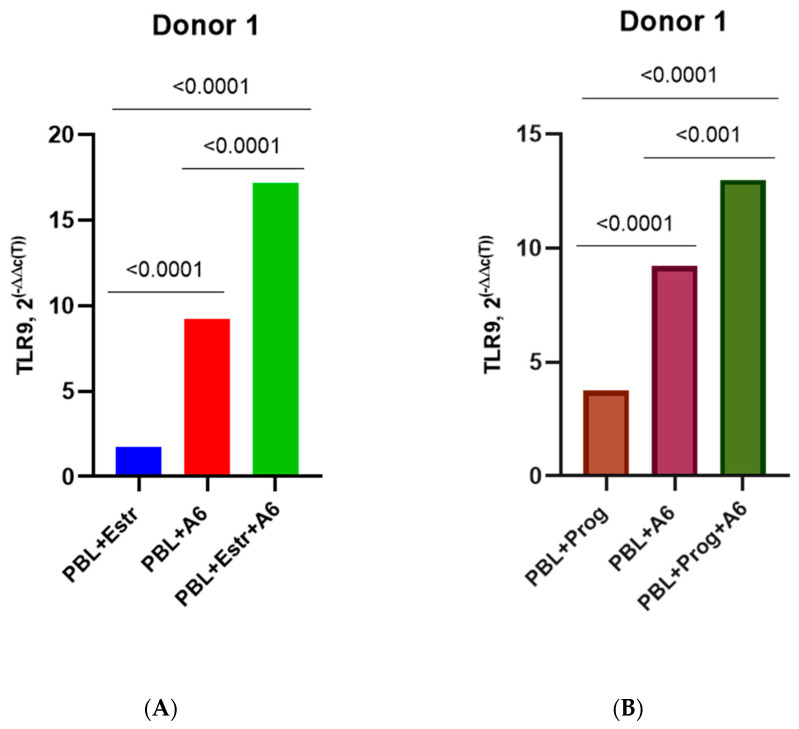

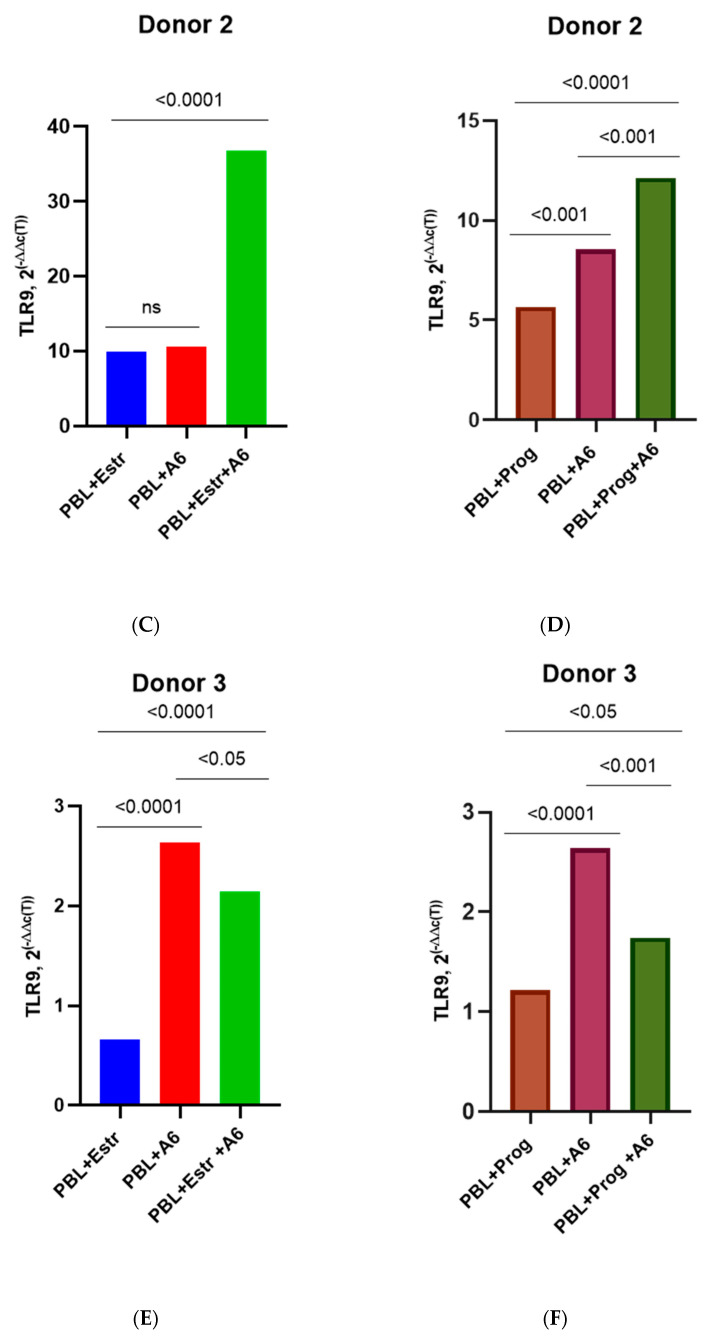

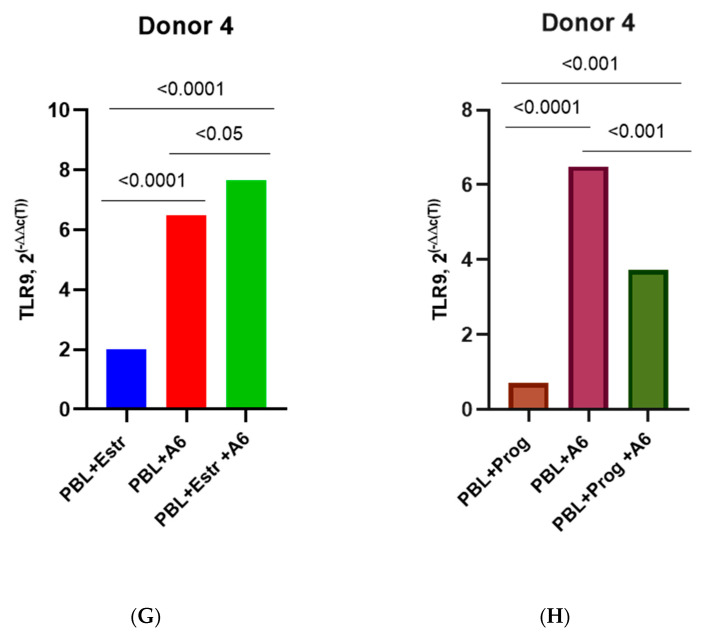
TLR9 expression in PBMCs of female donors infected with HIV-1 sub-subtype A6 in the presence of high-dose estradiol (5500 pg/mL) and progesterone (200 ng/mL): (**A**) Donor 1, estradiol; (**B**) Donor 1, progesterone; (**C**) Donor 2, estradiol; (**D**) Donor 2, progesterone; (**E**) Donor 3, estradiol; (**F**) Donor 3, progesterone; (**G**) Donor 4, estradiol; (**H**) Donor 4, progesterone. PBL, peripheral blood lymphocyte; Estr, estradiol; A6, HIV-1 sub-subtype A6; 2^(−∆∆C(T)), normalized expression coefficient. All data points are the averages of three culture wells run in triplicate; ns, no statistical difference, *p* > 0.05. For statistics, ANOVA was used.

In addition, estradiol upregulated the production of mRNA TLR9 in infected cells from Donors 1, 2, and 4 compared with infected PBMCs without hormones and uninfected PBMCs treated with hormones. Estradiol induced TLR9 expression in infected lymphocytes with the hormone by 1.9, 3.5, and 1.2 times (*p* ≤ 0.0001; *p* ≤ 0.0001; and *p* ≤ 0.05, respectively) compared with infected cells without the hormone and by 10.1 and 3.8 times (both *p* ≤ 0.0001) compared with uninfected PBMCs treated with hormones (Figure 6A,C,G). For Donor 3, a slight decrease in TLR9 expression under the influence of estradiol was observed in infected cells compared to infected PBMCs without the hormone (Figure 6E).

Progesterone induced TLR9 expression in the infected lymphocytes of Donors 1 and 2 by 1.4 times (both *p* ≤ 0.0001) compared with infected cells without the hormone and by 3.5 and 2.1 times (both *p* ≤ 0.0001) compared with uninfected PBMCs treated with hormones (Figure 6B,D). However, progesterone reduced TLR9 production in the infected PBMCs of Donors 3 and 4 compared with infected cells without the hormone but also upregulated TLR9 expression by 1.5 and 5.3 times (*p* < 0.05 and *p* < 0.0001) compared to uninfected PBMCs treated with progesterone (Figure 6F,H).

**Figure 7:**
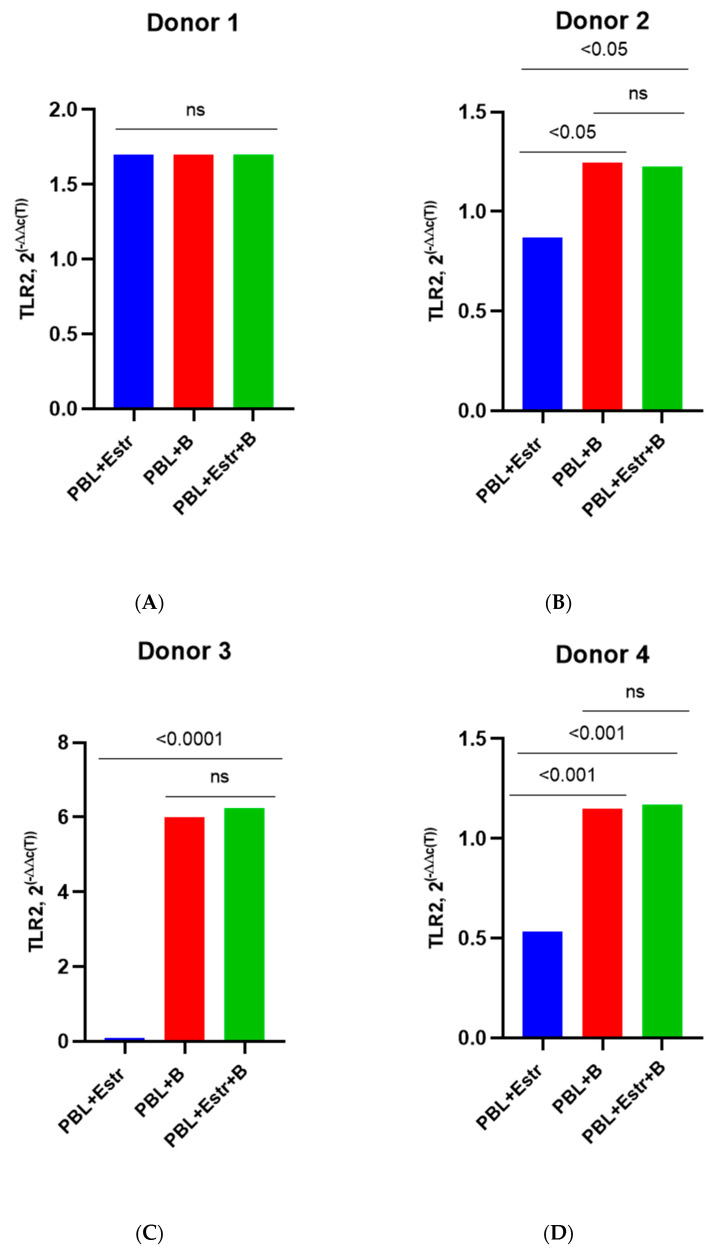
TLR2 expression in the PBMCs of female donors infected by HIV-1 subtype B in the presence of high-dose estradiol (5500 pg/mL): (**A**) Donor 1; (**B**) Donor 2; (**C**) Donor 3; (**D**) Donor 4. PBL, peripheral blood lymphocyte; Estr, estradiol; B, HIV-1 subtype B; 2^(−∆∆C(T)), normalized expression coefficient. All data points are the averages of three culture wells run in triplicate; ns, no statistical difference, *p* > 0.05. For statistics, ANOVA was used.

Unlike in PBMCs infected with the sub-subtype A6, there was no difference in the expression of TLR2 under the influence of estradiol (high dose 5500 pg/mL) in lymphocytes infected with HIV-1 subtype B (Figure 7A–D).

**Figure 8:**
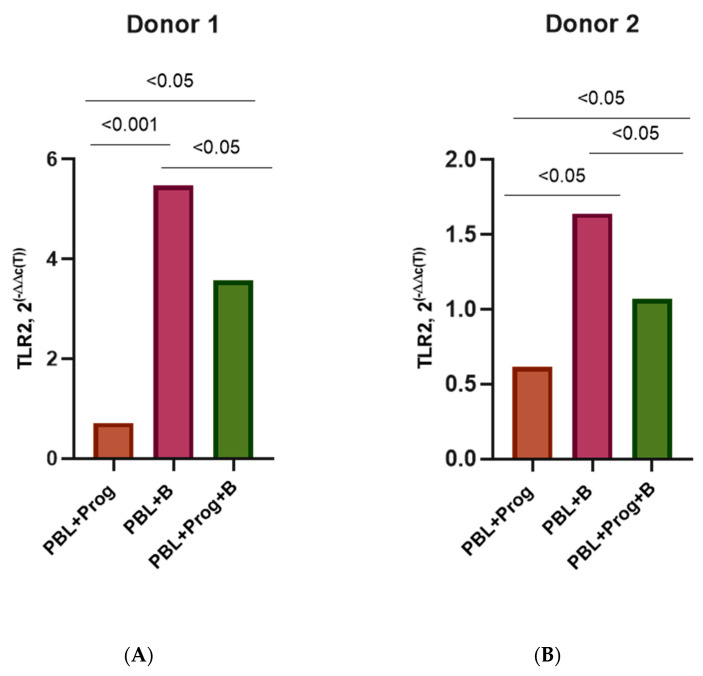

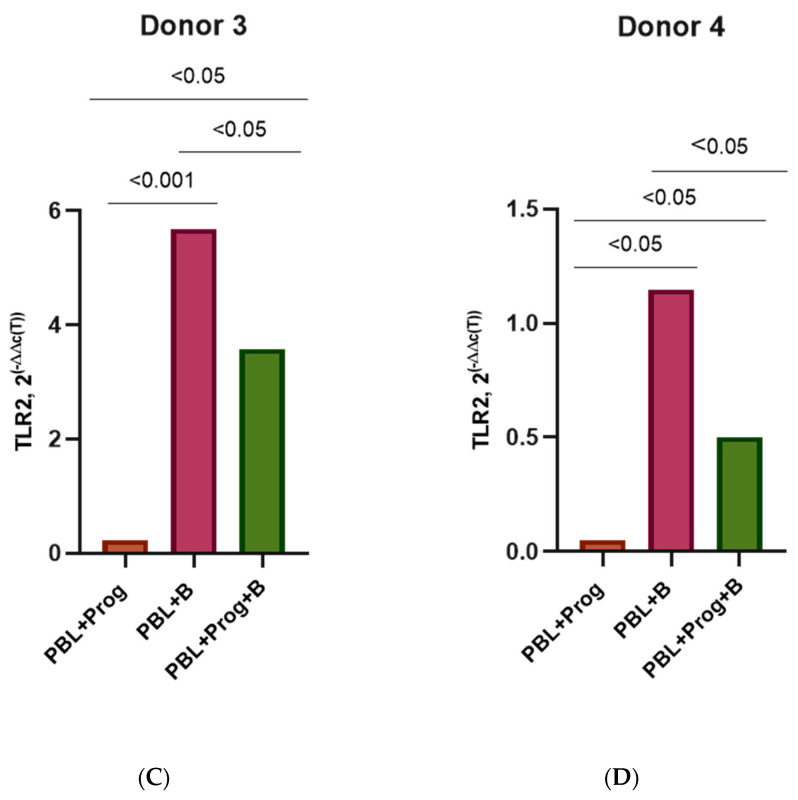
TLR2 expression in the PBMCs of female donors infected by HIV-1 subtype B in the presence of high-dose progesterone (200 ng/mL): (**A**) Donor 1; (**B**) Donor 2; (**C**) Donor 3; (**D**) Donor 4. PBL, peripheral blood lymphocyte; Estr, estradiol; B, HIV-1 subtype B; 2^(−∆∆C(T)), normalized expression coefficient. All data points are the average of three culture wells run in triplicate. For statistics, ANOVA was used.

However, under the influence of a high dose of progesterone, there was an average 1.5–1.7-fold decrease (*p*< 0.05) in TLR2 expression in infected PBMCs compared with infected PBMCs without the hormone (Figure 8A–D).

**Figure 9:**
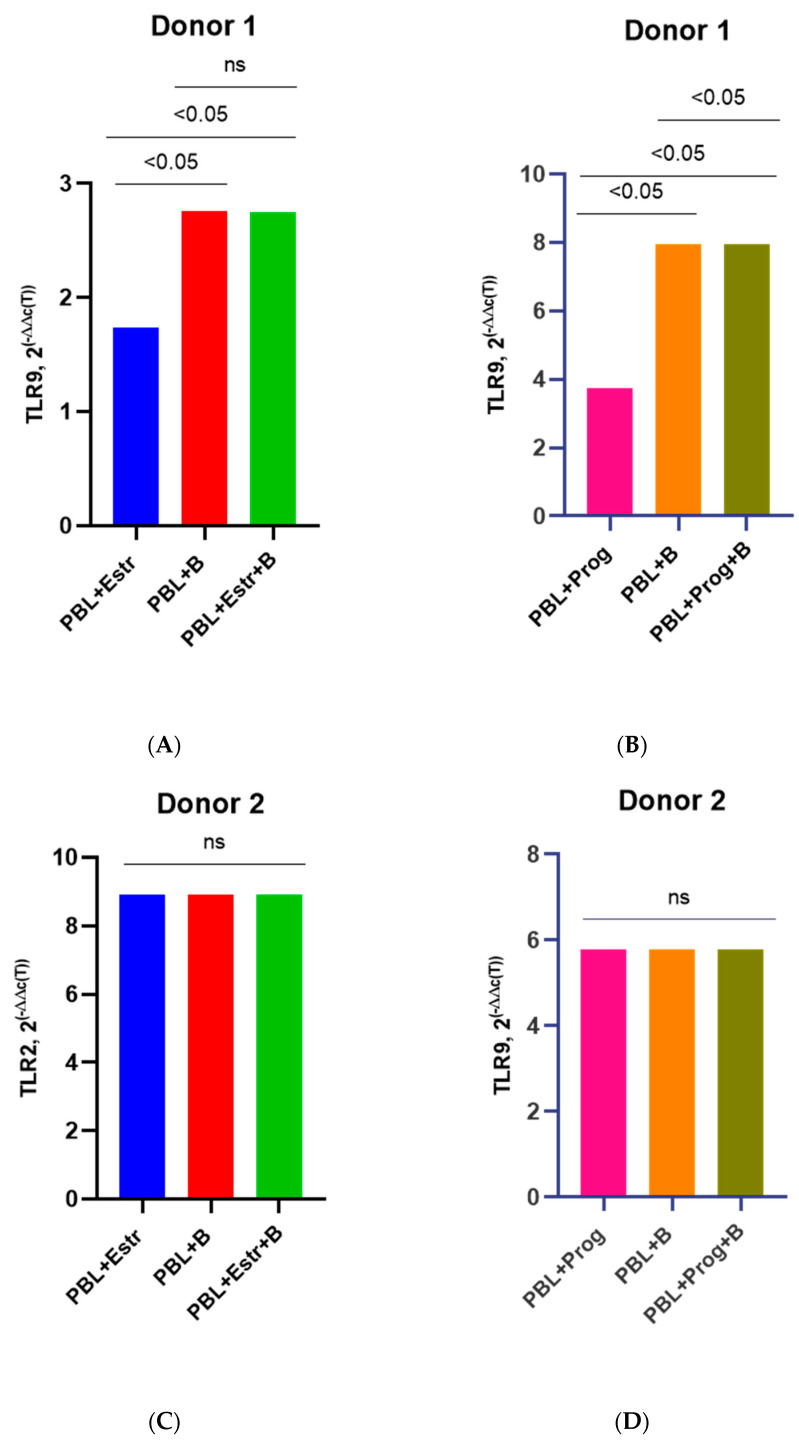

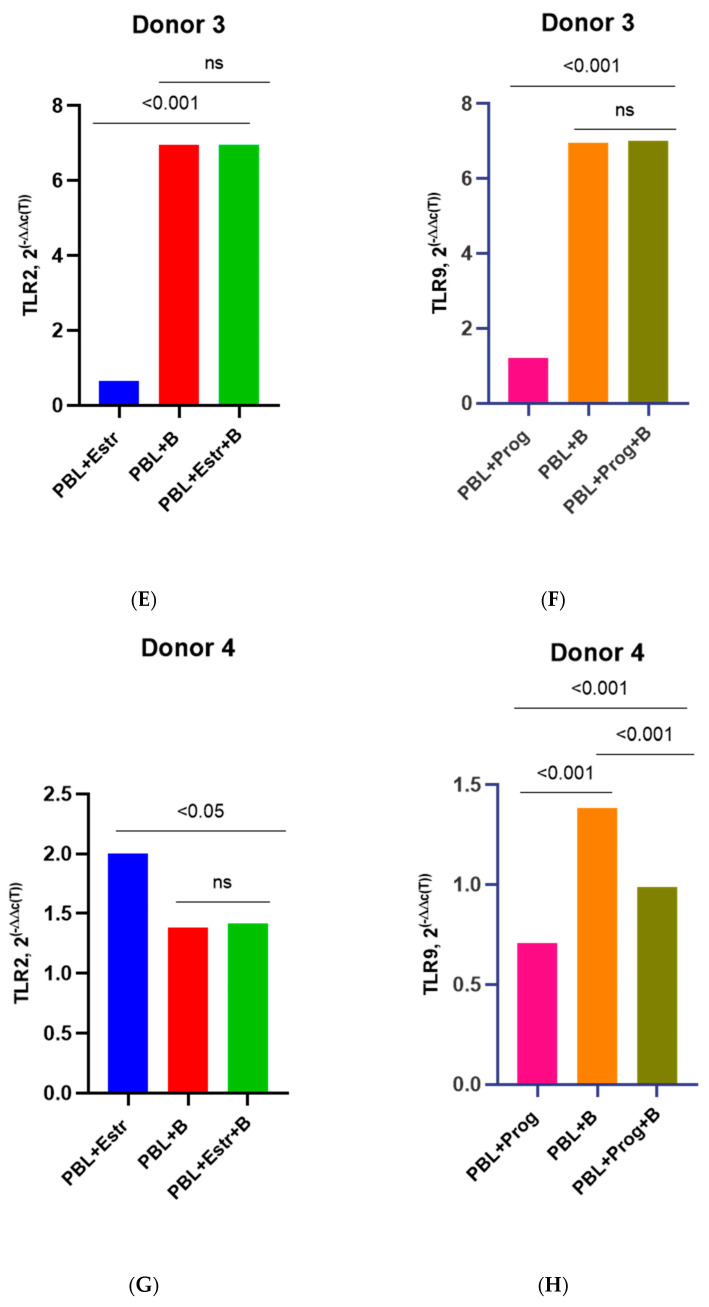
TLR9 expression in the PBMCs of female donors infected with HIV-1 subtype B in the presence of high-dose estradiol (5500 pg/mL) and progesterone (200 ng/mL): (**A**) Donor 1, estradiol; (**B**) Donor 1, progesterone; (**C**) Donor 2, estradiol; (**D**) Donor 2, progesterone; (**E**) Donor 3, estradiol; (**F**) Donor 3, progesterone; (**G**) Donor 4, estradiol; (**H**) Donor 4, progesterone. PBL, peripheral blood lymphocyte; Estr, estradiol; B, HIV-1 subtype B; 2^(−∆∆C(T)), normalized expression coefficient. All data points are the averages of three culture wells run in triplicate; ns, no statistical difference, *p* > 0.05. For statistics, ANOVA was used.

As for the expression of TLR9, high doses of hormones did not affect their production in any way. There was no difference in the level of TLR9 expression in cells infected with subtype B in the absence of hormones or in their presence (Figure 9A–G). The only exception was Donor 4. Under the influence of progesterone in infected PBMCs, a 1.4-fold decrease (*p* < 0.05) in TLR9 production was noted compared to that of infected cells without the hormone (Figure 9H).

**Figure 10:**
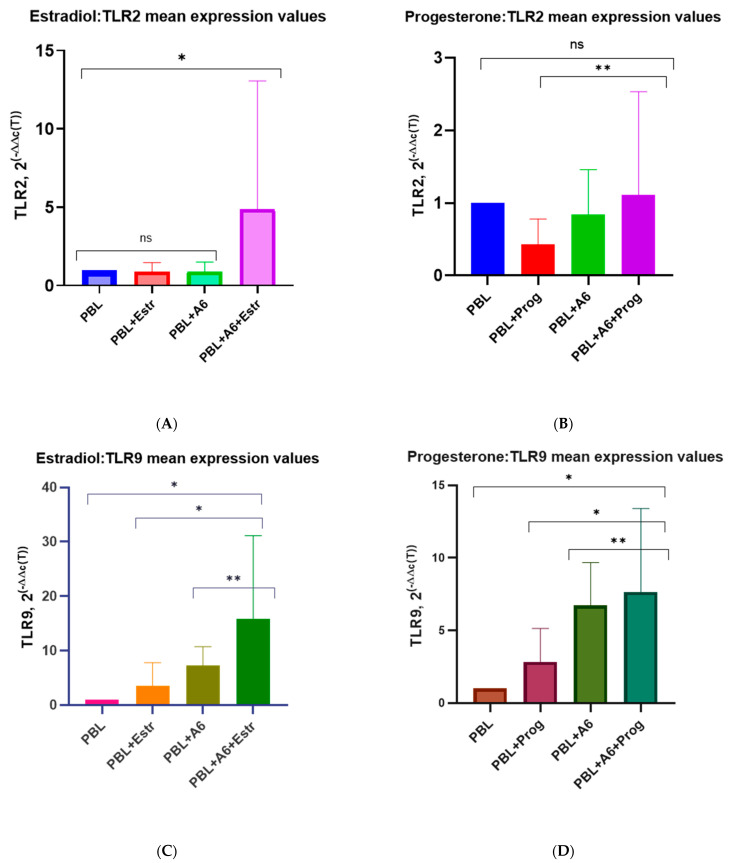
TLR2 and TLR9 median expression in the PBMCs of female donors in the presence of high-doses of estradiol (5500 pg/mL) and progesterone (200 ng/mL). PBL, peripheral blood lymphocytes; Estr, Estradiol; A6, HIV-1 sub-subtype A6. (**A**) TLR2 expression in the presence of β−estradiol; (**B**) TLR2 expression in the presence of progesterone; (**C**) TLR9 expression in the presence of β−estradiol; (**D**) TLR9 expression in the presence of progesterone; 2^(−∆∆C(T))—normalized expression coefficient. Results are expressed as the mean ± SD. All data points are the averages of three cultures wells run in triplicate. An asterisk (*) over the bars indicates a significant difference, *p* ≤ 0.01. An asterisk (**) over the bars indicates a significant difference, *p* ≤ 0.05. ns, no statistical difference, *p* > 0.05. For statistics, ANOVA was used.

The grouped results for all four donors are presented in Figure 10.

## 4. Discussion

Since it is well known that susceptibility to HIV-1 infection largely depends on genetic factors and can vary from person to person [51,52,53,54,55], first of all, it was decided to study the potential effect of hormones on viral replication in established cell lines, which would make it possible to create the same conditions for cell infection. For that purpose, the MT-4 and Jurkat cell lines were selected. MT-4 cells are very susceptible to cell-to-cell infection; thus, viral replication and release in these cells occur faster than in other T-cell lines [56], which is especially important when cells are infected with HIV-1 primary isolates rather than laboratory strains. However, since MT-4 cells carry human T-cell leukemia virus type 1 (HTLV-1) [57], which could potentially affect the results of the experiment, we also used the Jurkat lymphoblastoid cell line permissive to HIV-1 infection. Similar results were obtained when MT-4 cells and Jurkat cells were infected with HIV-1. The low concentrations of hormones had no effect on the viral production of any of the three subtypes. However, with high concentrations of estradiol and progesterone, an increase in viral replication was noted when cells were infected with the A6 sub-subtype and the recombinant CRF02_AG variant. The obtained results are confirmed by the work of other researchers, who have shown that estrogen and progesterone induce the production of HIV-1 [22,58,59].

In order to create conditions of cell infection in vitro as similar as possible to the conditions in vivo, the effect of hormones on the replicative kinetics of HIV-1 of various subtypes was studied in PBMCs isolated from the blood of female donors. The levels of HIV-1 replication among subtypes varied somewhat depending on the donor, but in general, this indicator was comparable to the production of the virus in the MT-4 and Jurkat cell lines. As in the case of the infection of established cell lines, high concentrations of estradiol and progesterone enhanced the replication of sub-subtype A6 and recombinant CRF02_AG but did not affect the production of subtype B. The data we obtained are not entirely consistent with the results of Ragupathy et al., who noted a decrease in the replicative activity of subtype A virus with high concentrations of hormones [60]. This may be because the cells were infected with other sub-subtype A than A6 (in the work of Ragupathy et al., it is not specified which particular sub-subtype the cells were infected with), and it is known that genetic variability within HIV-1 subtypes is 15–20%, which leads to different phenotypic properties of the virus [61,62,63,64]. At the same time, Ragupathy et al. observed a similar pattern in PBMCs isolated from the blood of a female donor infected with HIV-1 subtype A, which we also identified: high concentrations of estradiol and progesterone upregulated the replication of this subtype [60]. This once again confirms that the susceptibility of cells to virus infection is largely influenced by the genetics of the host. In this study, there was no difference in the replication of HIV-1 subtype B under the influence of hormones noted by other researchers [36,37], which may be explained by the differences in the phenotypic properties of the isolates.

In general, the observed discrepancy in the virus production by isolates of various HIV-1 subtypes under the influence of hormones can largely be explained by the fact that HIV-1 subtypes differ significantly, genetically speaking. Within group M, the genetic variability between subtypes is 15% for the *gag* gene and 25–35% for the *env* gene [65]. It is known that isolates of this group demonstrate a certain degree of genetic variability, particularly in the V3 loop region [65,66,67]. Inter-subtype analysis has also shown substantial differences in *vif* and *nef* genes and long terminal repeat (LTR) regions. Additionally, it is important to take into account the tropism of the isolate. These genetic variations inevitably lead to differences in the biological properties of HIV-1 variants. It is also necessary to consider that, in addition to subtype divergence and interhost variability, there is intra-host variability, which accounts for 6–19% [64]. Considering this, it would be of great interest to study the replication features of several isolates of the same HIV-1 subtype in PBMCs derived from one donor.

The mechanisms by which sex steroid hormones enhance viral replication and affect HIV infection are not fully understood. Some researchers have shown that hormones can affect the expression of CCR5 and CXCR4 co-receptors [22,59,68,69,70]. It has been shown that levonorgestrel (LNG) and DMPA increase the expression of CCR5 co-receptors on the surface of peripheral T cells [22,71]. Prakash et al. demonstrated an increased expression of both CCR5 and CXCR4 co-receptors under the influence of steroid hormones [72]. Other researchers showed that progesterone reduced the expression of CCR5 co-receptors and increased the expression of CXCR4 co-receptors on the surface of PBMCs of HIV-negative women [68]. It was also found that, in women at different stages of the menstrual cycle, elevated progesterone levels were associated with increased levels of CCR5 and CXCR4 expression in the tissues of the genitals [69]. A direct correlation was found between increased progesterone levels during pregnancy and an increased expression of CCR5 co-receptors both on the surface of T cells and in the genital tissues [69]. The obtained data suggest that estradiol and progesterone may have an effect on HIV-1 at the receptor level. Taking this into account, we studied the possible enhancement in the expression of those receptors under the influence of estradiol and progesterone, which could explain the increase in viral replication, since these receptors play key roles in the infection of cells with HIV-1 [73,74,75]. However, we found no difference in the expression levels of CCR5 and CXCR4 co-receptors on the cell surface, either in the presence of hormones or without them. Similar results were obtained by other researchers, who showed no increase or decrease in the expression of those co-receptors under the influence of hormones in vitro, both in established cell lines and PBMCs [60].

It is likely that steroid hormones alter the expression of other co-receptors that are also responsible for the penetration of the virus into cells. For example, it has been shown that about 57% of HIV-1 isolates use cell infection chemokine receptor CCR8, which is expressed on the surface of T-lymphocytes [76]. It has also been shown that, along with CCR5 and CXCR4 co-receptors, HIV-1 can use the chemokine receptors CCR2, CXCR3, CX3CR1, CCR3, CXCR6 (STLR33/Bonzo), and CXCR7 (ACKR3) [53,77,78,79,80,81,82]. Further, data have emerged indicating that CXCR7 is used by a significantly larger number of HIV-1 isolates than was previously assumed [81,83,84]. In addition, it has been shown that the interactions of estrogen receptor-α (ERα) with G-proteins, various membrane receptors, and signaling molecules promote the intracellular activation of mitogen-activated protein kinase (MAPK) and protein kinase B signaling pathways, thereby inducing transcription from the long terminal repeats (LTRs) in infected cells, which enhances virus replication [85]. Thus, it is likely that estradiol both affects the expression of alternative co-receptors and modulates HIV transcription.

Since the detected increase in viral replication in HIV-1-infected cells in the presence of hormones was not induced by the enhanced production of CCR5 or CXCR4 co-receptors, we decided to investigate the possible effect of hormones on the expression of Toll-like receptors (TLRs), which, according to recent studies, might also play a key role in HIV infection [86,87,88,89]. It is assumed that, besides the direct intracellular interaction of TLRs with viral ligands due to infection by HIV-1 cells, TLRs can also play an important role in the sensibilization of molecules that contain TLR ligands and are released as a result of the lysis of infected cells, thus triggering TLR-dependent paracrine effects [86]. We chose to study TLR2 and TLR9 because of the important role they play in HIV-1 infection. TLR2 is a cell membrane TLR that increases HIV-1 replication by promoting better penetration of the virus into cells through cortical actin remodeling and creating a more favorable environment for the expression of viral genes [90]. TLR9 is an intracellular TLR that is expressed within the host cells and is associated with a higher HIV-1 RNA load. Particularly, it was shown that the specific polymorphism in TLR9 (TLR9 1635AA genotype) was associated with a higher HIV-1 RNA load, though the underlying mechanisms by which TLR9 influences the viral load are still not understood [91]. Since the study did not reveal the effect of hormones on the replication of HIV-1 subtype B, first of all, it was of interest to study the expression of the TLRs genes in PMBCs infected with the HIV-1 A6 sub-subtype, as the replication of that particular sub-subtype was impacted by the hormones (taking into account the similarity of the biological properties of A6 and recombinant CRF_02AG; the expression of TLRs in PBMCs infected with CRF_02AG was not studied). Estradiol and progesterone have been found to upregulate TLR2 and TLR9 expression in the infected PBMCs of female donors to varying degrees. In infected lymphocytes from three donors in the presence of estradiol, an increased expression of TLR2 mRNA by an average of 1.7 times was observed compared with HIV-1 infected PMBCs cultured in the absence of the hormone and by 4.3 times compared to uninfected cells treated with estradiol. For one donor, there was no difference in the expression of the receptor in infected cells without the hormone and in infected cells in the presence of the hormone. This can largely be explained by the interhost genetic factors conditioning the specific virus–host relationship, as it is known that interhost variability accounts for 2–5% [64]. While a statistically significant increase in TLR2 production (by 1.9 times) in infected PMBCs in the presence of progesterone was noted for only one donor, and enhanced expression was observed compared to uninfected PBMCs treated with progesterone (by 6.8 times, on average) for two donors, it can be assumed that progesterone might also induce TLR2 expression, depending on host factors. Thus, the observed increase in HIV-1 A6 replication in the presence of hormones could be partially explained by the increased expression of TLR2, which has been shown to indirectly facilitate the entry of the virus into cells and promote its replication [90,92]. The observed difference in TLR2 expression in infected PBMCs from different female donors once again indicates that the genetics of the host play an important role in the susceptibility of cells to HIV-1 infection.

As in the case of TLR2 production, the expression of TLR9 mRNA varied in PBMCs from different donors, but in general, in the presence of hormones, TLR9 expression in HIV-1-infected PBMCs was upregulated compared with uninfected PBMCs treated with estradiol and progesterone, by almost three to four times. At present, a lot of data indicating that various variants of TLR9 affect the viral load and, ultimately, the outcomes of HIV infection have already been accumulated [86]. It has been shown that a certain polymorphism in the TLR9 gene is associated with increased viral replication [91,93,94]. In this work, we did not study this issue, but it is possible that TLR9 polymorphisms could explain the different levels of its production in PBMCs from various donors.

We have not detected enhanced viral production under the influence of hormones in cells infected with subtype B. At the same time, we also did not detect an increase in the expression of TLR2 and TLR9 in PBMCs infected with subtype B in the presence of hormones. This fact indirectly confirms that increased viral replication and increased TLRs expression are interrelated. Of course, taking into account the intra-clade genetic diversity, it should be further studied whether this trend is characteristic only for this particular isolate or for all isolates of subtype B.

The obtained data on TLR production in lymphocytes infected with HIV-1 sub-subtype A6 in vitro allow us to conclude that one of the factors that enhance viral replication in PBMCs from female donors in the presence of estradiol is the hormone-induced increased expression of mRNA TLR2 and TLR9. This is consistent with the results of other authors, who showed that the activation of TLR2 and TLR9 production in HIV-1-infected cells led to the enhanced production of the virus [95]. As for the effect of progesterone on the expression of TLR2 and TLR9 in HIV-infected cells, its role is somewhat contradictory given that, in PBMCs from some donors, there was definitely an increase in the expression of TLRs under its influence, and in PBMCs from others, on the contrary, there was a slight decrease in the expression of mTLR2 and mTLR9. Apparently, in this case, as mentioned above, interhost genetic factors and intra-host variability play an important role. Studies on the expression of TLRs show that their production varies significantly in different individuals, especially in people living with HIV [88]. Undoubtedly, further study is required of TLR2 and TLR9 expression in HIV-infected cells under the influence of progesterone on a larger number of cells from various female donors, but, unfortunately, here, we face certain difficulties associated with the donor’s blood.

Additionally, it is likely to assume that the augmented viral replication under the influence of progesterone in HIV-1 infected cells, in which there was no increase in the expression of TLR2 and TLR9, might be caused by the enhanced production of other TLRs. For example, lately, there has been evidence that TLR7 may play an important role in retroviral infections, since retroviruses have single-stranded RNA (ssRNA) genomes, and TLR7 binds and reacts to ssRNA in endosomal compartments [86]. Recent studies have shown that, despite the fact that the level of TLR7 expression in T-cells was rather low, HIV-1 induced the TLR7-dependent expression of the anergic gene in T-cells, thus helping HIV-1 to escape elimination [86]. Further, studies evaluating the associations of the TLR variant with the presence of HIV-1 infection showed that the TLR7 32A/T variant was more often detected in HIV-infected women compared to uninfected women [94]. There is also evidence that there may be an increase in TLR4 production in viral infections [88]. The study of the production of other TLRs is the subject of future research. However, in any case, the obtained data clearly indicate that, under the influence of progesterone, as well as estradiol, an increase in the replication of HIV-1 sub-subtype A6 is detected.

It is known that physiological hormone levels fluctuate in the peripheral blood and reproductive organs of women during the menstrual cycle. A lot of data have accumulated, indicating that fluctuations in hormone levels play an important role in the body’s immune response to HIV infection and susceptibility to infection [16,96,97,98,99]. A number of researchers have found that estradiol and progesterone regulate HIV-1 replication in PBMCs, increasing its replication during the follicular phase and reducing it during the luteal phase [16,99]. Thus, the highest frequency of HIV-1 shedding is observed immediately after menstruation, which increases the risk of HIV transmission from a woman to her sexual partner [100]. A higher concentration of HIV-1 RNA is found in cervicovaginal secretions during pregnancy, and elevated concentrations of estrogen and progesterone during pregnancy correlate with an increased severity of the infectious process [16].

## 5. Conclusions

The study demonstrated that female sex hormones that are part of hormonal contraceptives may have differing effects on the virus replication of various HIV subtypes. In our experiments using established cell lines and female PBMCs, we found that a high hormones concentration promoted the replication of HIV-1 sub-subtype A6 and recombinant form CRF02_AG, though it did not affect the replication of HIV-1 subtype B. Those changes in virus replication were not due to the altered CCR5 or CXCR4 co-receptors expression. It is likely that steroid hormones alter the expression of other alternative co-receptors, which are also responsible for the penetration of the virus into the cell. Additionally, the significant involvement of female sex hormones in the modulation of TLR gene expression in HIV-1-infected cells was shown—in particular, TLRs genes 2 and 9, which play an important role in HIV-1 infection. It was demonstrated that estradiol upregulated the production of TLR2, and the expression of TLR9 was upregulated by both estradiol and progesterone. We are aware that there are certain limitations of our work and believe that further studies should be conducted on a larger group of donors, and a larger number of different isolates of the same subtype should be studied. Due to the small number of donors, it is not entirely correct to draw general conclusions. However, based on the results obtained, it can be said with certainty that hormones can induce increased TLR expression in the PBMCs of female donors. Overall, the obtained findings highlight the need to take into account the possible impact of hormones on the enhancement of viral replication. Additionally, these findings set the direction for future research: to study the expression of alternative chemokine co-receptors under the influence of hormones in HIV infection and to study the production of other TLRs, such as TLR-4, -7.

## Data Availability

Not applicable.

## Supplementary Materials

The following supporting information can be downloaded at: https://www.mdpi.com/article/10.3390/pathogens12070880/s1, Table S1. Determination of toxic effects of β-estradiol and progesterone on MT-4 and Jurkat cells and PBMCs. Figure S1.Replication of HIV-1 (sub-subtypes A6, CRF02_AG, and B) in the presence of estradiol and progesterone in Jurkat cells. Figure S2. CCR5 and CXCR4 co-receptor expression in PBMCs of female donors in the presence of high doses of estradiol (5500 pg/mL) and progesterone (200 ng/mL), Day 7 post-infection.
