# Supplementary material for: Female Sex Hormones Upregulate the Replication Activity of HIV-1 Sub-Subtype A6 and CRF02_AG but Not HIV-1 Subtype B
Source: Pathogens. 2023 Jun 27;12(7):880. doi: 10.3390/pathogens12070880 (PMC10383583; doi:10.3390/pathogens12070880)
Supplement: Supplementary file 1 [file pathogens-12-00880-s001.zip › Supplemental Figure S1. Replication of HIV-1 in Jurkat.pdf]

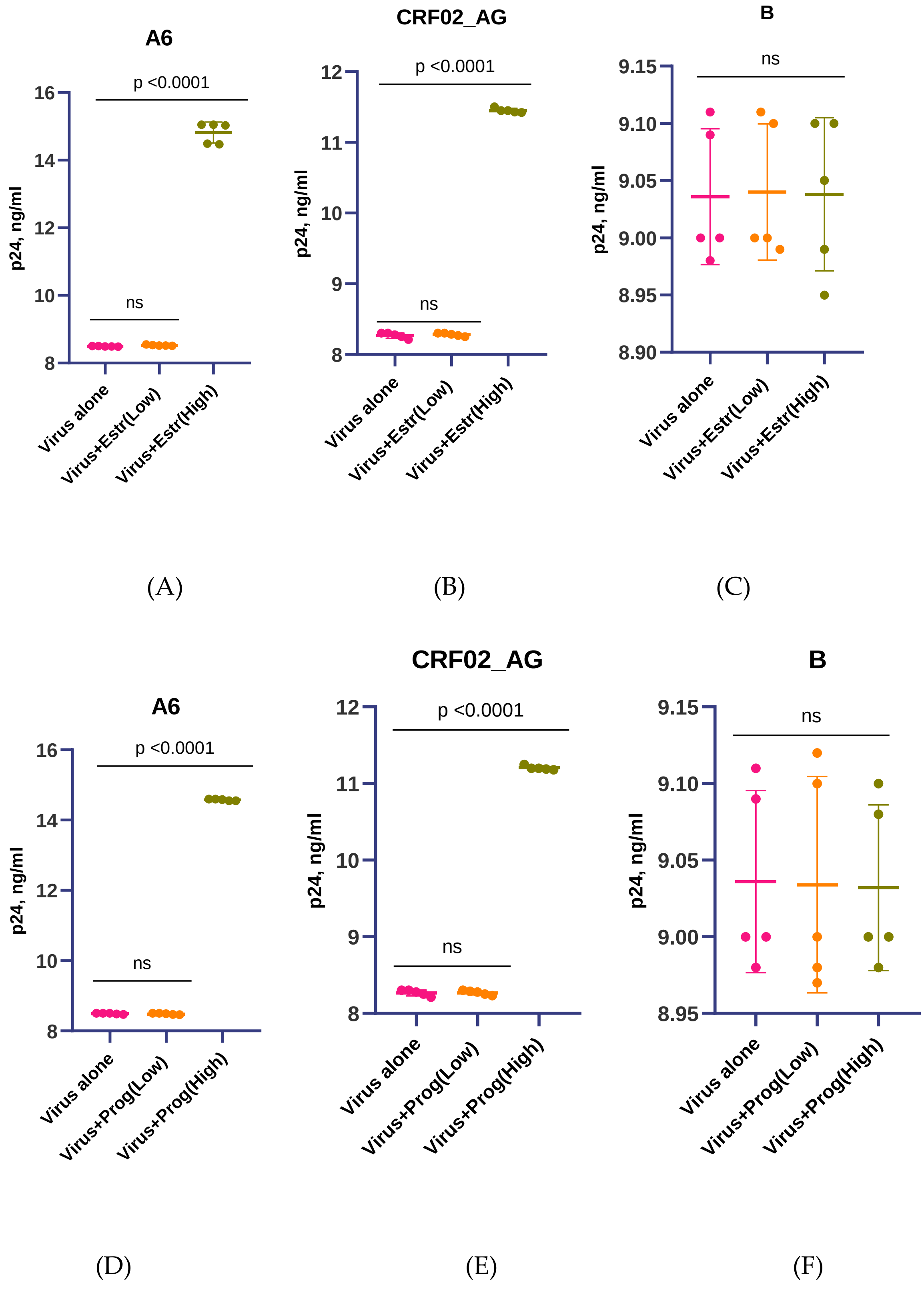

Supplemental Figure S1. Replication of HIV-1 (sub-subtypes A6, CRF02_AG, and B) in the presence of estradiol and progesterone in Jurkat cells: low-dose β-estradiol (250 pg/ml); high-dose β-estradiol (5500 pg/ml); low-dose progesterone (89 ng/ml); high-dose progesterone (200 ng/ml). (**A**) sub-subtype A6 + low/high-dose β-estradiol; (**B**) recombinant form CRF02_AG + low/high-dose β-estradiol; (**C**) subtype B + low/high-dose β-estradiol; (**D**) sub-subtype A6 + low/high-dose progesterone; (**E**) recombinant form CRF02_AG + high-dose progesterone; (**F**) subtype B + low/high-dose progesterone. Results are representative of 5 independent experiments. For each experiment, all data points are averages of three culture wells run in triplicate; ns indicates no statistical difference from control, $p > 0.05$. For statistics ANOVA was used.
