# Supplementary material for: Female Sex Hormones Upregulate the Replication Activity of HIV-1 Sub-Subtype A6 and CRF02_AG but Not HIV-1 Subtype B
Source: Pathogens. 2023 Jun 27;12(7):880. doi: 10.3390/pathogens12070880 (PMC10383583; doi:10.3390/pathogens12070880)
Supplement: Supplementary file 1 [file pathogens-12-00880-s001.zip › Supplemental Figure S2. CCR5 and CXCR4 co-receptor expression in PBMCs.pdf]

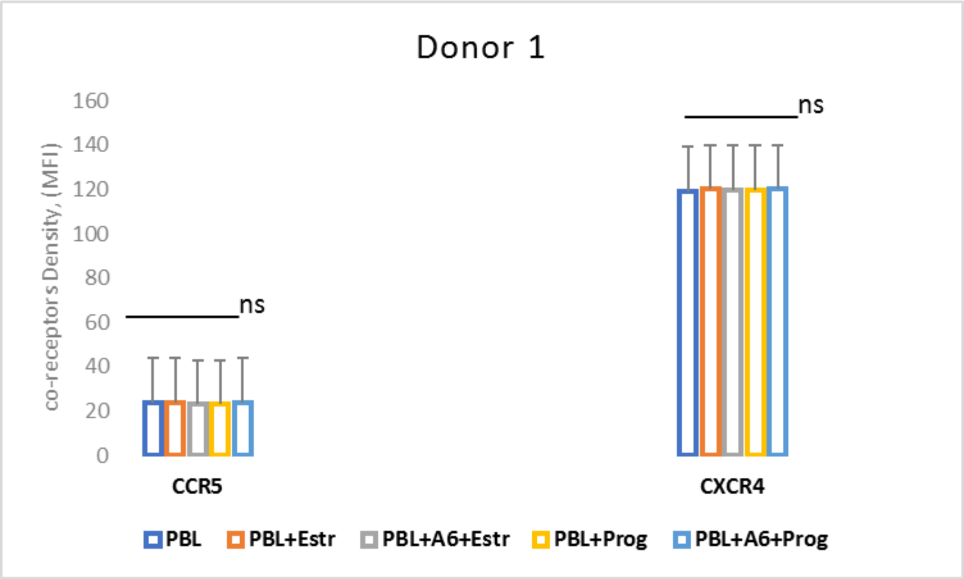

(A)

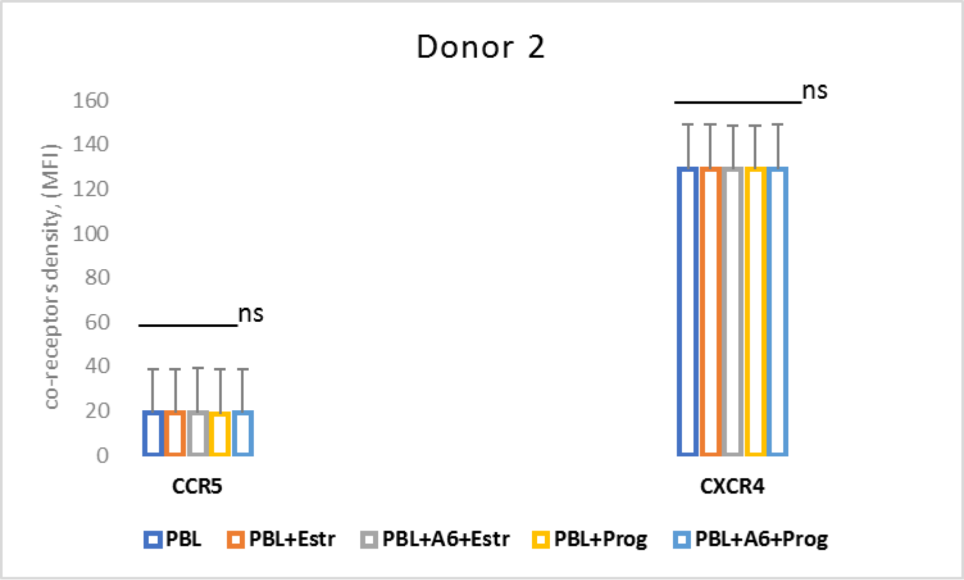

(B)

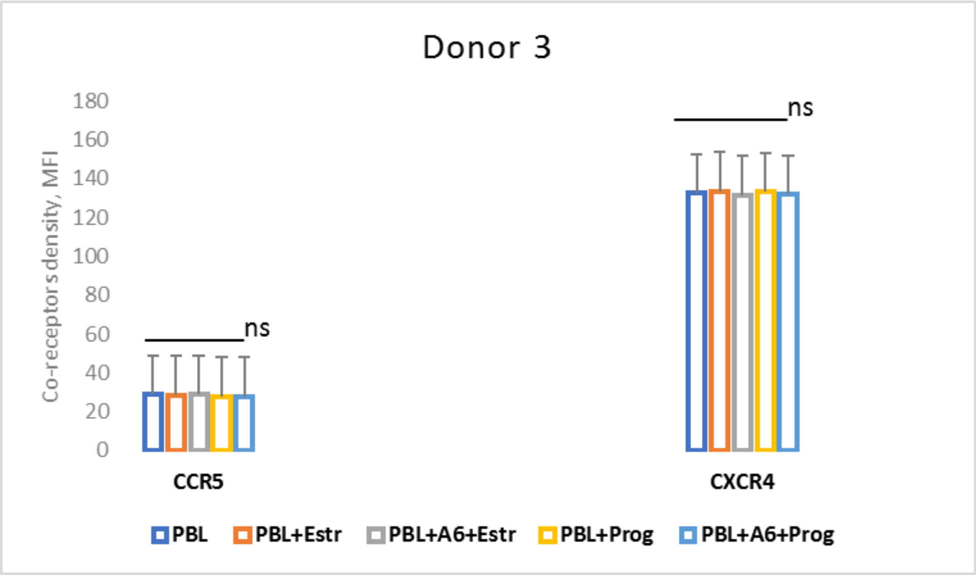

(C)

Fig.S1. Cont.

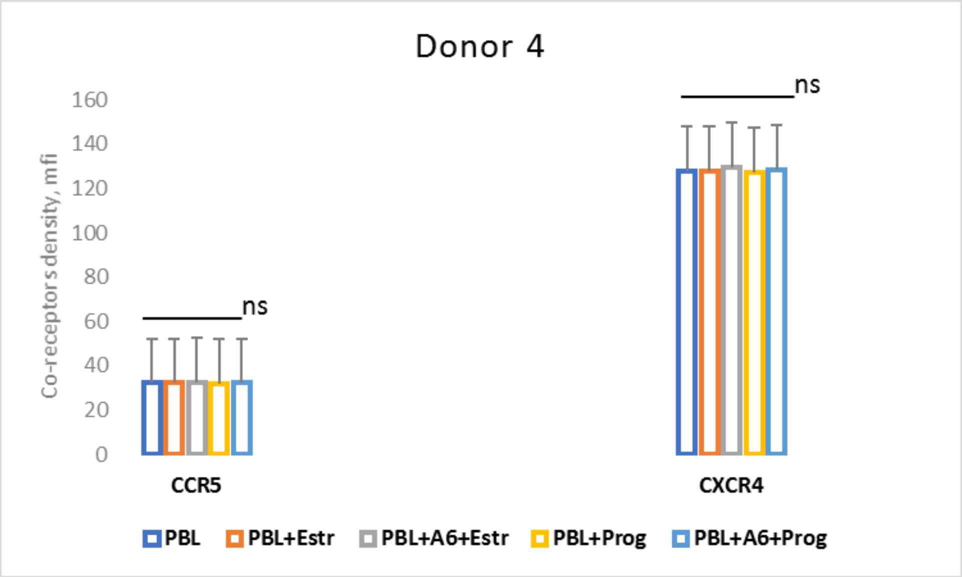

(D)

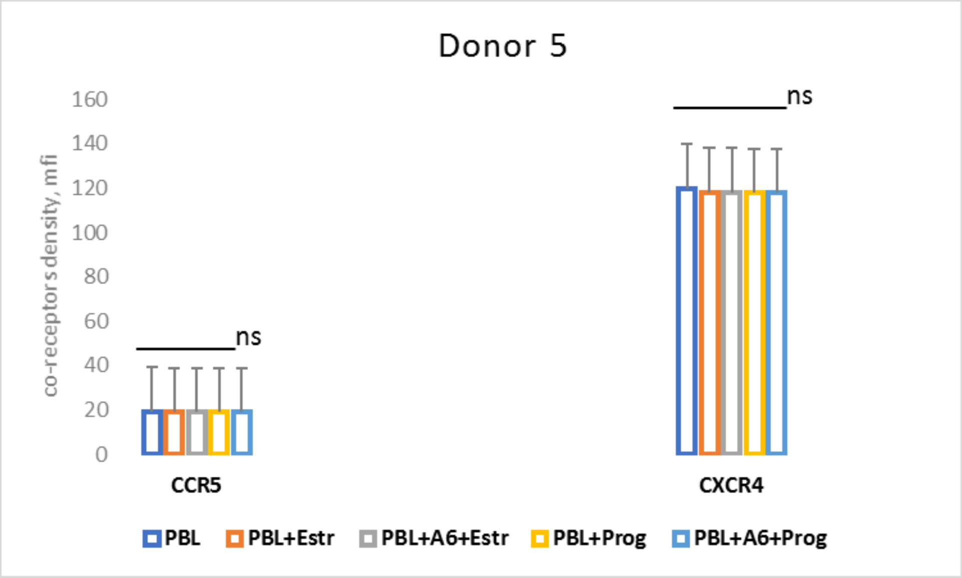

(E)

Supplemental Figure S2. CCR5 and CXCR4 co-receptor expression in PBMCs of female donors in presence of high doses of estradiol (5500 pg/ml) and progesterone (200 ng/ml), Day 7 post infection. (**A**) Mean density of CCR5 and CXCR4 in PBMCs of (**A**) Donor 1; (**B**) Donor 2; (**C**) Donor 3; (**D**) Donor 4; (**E**) Donor 5. PBL, peripheral blood lymphocyte; Estr, estradiol; Prog, progesterone; A6, HIV-1 sub-subtype A6. Results expressed as mean fluorescence index (MFI). ns indicates no statistical difference, $p \geq 0.05$.
