# Supplementary material for: Female Sex Hormones Upregulate the Replication Activity of HIV-1 Sub-Subtype A6 and CRF02_AG but Not HIV-1 Subtype B
Source: Pathogens. 2023 Jun 27;12(7):880. doi: 10.3390/pathogens12070880 (PMC10383583; doi:10.3390/pathogens12070880)
Supplement: Supplementary file 1 [file pathogens-12-00880-s001.zip › Supplemental Table S1.pdf]

Supplemental Table S1. Determination of toxic effects of β-estradiol and progesterone on MT-4 and Jurkat cells and PBMCs.

Hormone concentration	Percentage of viable cells (%)						
	MT-4	Jurkat	Donor 1	Donor 2	Donor 3	Donor 4	Donor 5
**β-estradiol** **with hormone/no hormone**							
250 pg/ml	100/100	98.8/99.8	97.9/97.7	98.5/98.5	98.3/98.3	87.9/88.2	87.7/87.9
5500 pg/ml	99.8/99.8	99.5/99.5	98.2/97.9	98.7/98.9	97.9/97.9	88.2/88.1	87.6/87.6
**Progesterone** **with hormone/no hormone**							
89 ng/ml	99.2/99.2	99.6/99.6	99.1/99.5	97.9/97.9	97.6/97.4	88.9/89.1	91.9/91.8
200 ng/ml	99.4/99.5	99.8/99.8	98.4/99.5	98.3/98.7	97.9/97.9	88.4/88.6	87.6/87.6

Data represent mean values of results of 3 independent experiments. For each experiment, all data points are averages of three culture wells run in triplicate. The numbers indicate the viability of cells in %.
